# Error in Author Affiliation

**DOI:** 10.1001/jamanetworkopen.2020.29291

**Published:** 2020-11-12

In the Invited Commentary titled “In Support of CDK4/6 Inhibitors—A Meta-analysis of Available Randomized Data,”^1^ published October 13, 2020, there was an error in the author affiliations. Dr Goldstein is located at the Department of Medical Oncology, Fox Chase Cancer Center, in Philadelphia, Pennsylvania. This article has been corrected.^1^
